# Expression and inactivation of glycogen synthase kinase 3 alpha/ beta and their association with the expression of cyclin D1 and p53 in oral squamous cell carcinoma progression

**DOI:** 10.1186/s12943-015-0300-x

**Published:** 2015-02-03

**Authors:** Rajakishore Mishra, Siddavaram Nagini, Ajay Rana

**Affiliations:** Centre for Life Sciences, School of Natural Sciences, Central University of Jharkhand, Ratu-Lohardaga Road, Brambe, Ranchi, 835205 Jharkhand INDIA; Department of Biochemistry and Biotechnology, Faculty of Science, Annamalai University, Annamalainagar 608 002, Tamil Nadu, INDIA; Department of Molecular Pharmacology & Therapeutics, Loyola University Chicago, 2160 South First Ave., Maywood, 60153 IL USA

## Abstract

**Background:**

The study aims to evaluate the expression and activity of glycogen synthase kinase 3 isoforms α/β (GSK3α/β) and to assess their oncogenic potential through a correlation with the expression of cyclin D1 and p53 in oral cancer.

**Methods:**

The expression of total and phosphorylated GSK3α/β as well as cyclin D1 and p53 together with their interaction were assessed in human oral cancer tissue samples, apparently normal adjacent tissues, benign tumor samples, premalignant lesions and healthy normal tissues (total 179) using various methods, such as immunohistochemistry, Western blot assays, immunoprecipitation and RT-PCR analysis.

**Results:**

The expression of GSK3β was significantly higher relative to GSK3α indicating the greater role of the β isoform in oral cancer. Among various types of oral cancers, OSCC (of the lip and tongue) showed elevated expression of GSK3α/β, and the expression was correlated with disease progression. The increased expression of pS^21^GSK3α and pS^9^GSK3β not only correlated positively with cyclin D1 and p53 expression in tongue cancer progression but a gradual shift of their expression from the cytoplasmic to the nuclear compartment and overall disease severity was also observed. The interaction of GSK3β-cyclin D1 and the positive correlation of pS^9^GSK3β and the transcription of cyclin D1 were observed.

**Conclusions:**

These results demonstrate that the inactivation of GSK3β is an important event in OSCC and can be used as a marker for assessing disease severity and may be exploited for therapeutic intervention.

## Introduction

Oral cancer is the sixth most common cancer in the world, and its incidence varies in different ecogeographic regions [1,2]. While tobacco smoking and alcohol consumption are major risk factors for oral cancer in the western population, betel quid chewing with tobacco is recognized as the predominant contributor to oral cancer prevalence in Southeast Asia [3]. The high incidence of oral cancer in the Jharkhand state in the eastern part of India may be attributed to use of locally made alcoholic beverages, such as Mohua prepared from the flowers of the mahua plant, and Hadia prepared from fermented cereals, in addition to tobacco chewing habit.

Glycogen synthase kinase 3, a serine/threonine kinase involved in multiple physiological processes is a highly conserved and ubiquitously expressed member of the CMGC family of protein kinases [4]. To date, two members of the mammalian GSK3 family (α and β) are known. GSK3α/β plays a major role in epithelial cell homeostasis [5]. GSK3 proteins usually have three domains, a small N-terminal domain, a slightly larger C-terminal domain and a predominant middle kinase domain. In addition to these domains, a nuclear localization sequence has also recently been identified [6]. Its paradoxical role as a tumor suppressor or a tumor promoter is actively under investigation in various neoplastic diseases [7]. GSK3 is a constitutively active enzyme in normal cells and undergoes rapid inhibition by stimuli. The activity of GSK3 is inhibited upon phosphorylation at Ser^21^ of GSK3α and at Ser^9^ of GSK3β [8]. GSK3 is a key suppressor of the canonical Wnt signaling pathway including β-catenin [9] and various other oncogenic transcription factors (OTFs), such as NFκB, AP-1, c-Myc and p53, which are involved in cell proliferation [10].

Cyclin D1, a proto-oncogene, is an important regulator of G1 to S phase progression in many different cell types [11]. Together with its binding partners cyclin dependent kinase 4 and 6 (CDK4 and CDK6), cyclin D1 forms active complexes that promote cell cycle progression [12]. Cyclin D1 is important for the development and progression of several cancer types, including that of oral epithelial cancer that occurs by the transformation of the buccal mucosa causing oral squamous cell carcinoma (OSCC) [13]. Overexpression of cyclin D1 protein is frequently the result of its deregulation at the post-translational level. Active GSK3α/β phosphorylates cyclin D1, leading to its degradation [14]; thus, suppressing signals that inactivate GSK3α/β causes epithelial cancer [15]. Alternatively, p53 is a well-known tumor suppressor protein that is widely reported in human cancer. Wild type p53 maintains genomic integrity through the induction of cell cycle and cell death regulatory genes in response to DNA damage [16]. Although mutational inactivation of p53 has been reported in nearly half of the oral cancer population, in the subpopulation of OSCC cases without p53 mutations the mechanism of p53 inactivation is still far from clear [17]. p53 activity is regulated by active GSK3β, due to either a physical association or phosphorylation and post-translational modifications [18].

In the present study, an investigation was performed to assess the expression of GSK3α/β in various stages of oral tumor progression. The activity of GSK3α/β was also assessed by detecting its site-specific phosphorylation in various oral cancer samples, more elaborately in oral tongue SCC (OTSCC) samples. The protein interaction of GSK3α/β with cyclin D1 in various oral tumors was determined, and the inactivation status of GSK3α/β was correlated with the expression of pro-cell cycle promoting cyclin D1 and with the expression of p53 in a group of random samples. The data suggest that the inactivation of GSK3, especially GSK3β, might be related to oral cancer progression and might fuel the transcription of cyclin D1. These pathways may be targeted to treat this deadly disease.

## Materials and methods

### Patients and tissue samples

A total of one hundred seventy-nine (n = 179) different human oral tumors and control samples, which include tissue microarray (TMA, OR802 and OR601a from US Biomax) samples (n = 140) and freshly collected human oral tumor and control samples (n = 39), were analyzed. The fresh samples of twenty-seven oral tumor samples, six normal samples and six PMLs samples (thick leukoplakia n = 3; OSMF n = 3) were collected from local hospitals, nursing homes and clinics near the Ranchi area. These samples were collected after obtaining informed consent from the patients, and the use of human samples was approved by the Institutional Human Ethical Committee of CUJ. The samples of normal and PMLs were obtained from patients without cancer undergoing nononcologic surgical procedures. The collected samples were divided into two pieces and stored in liquid nitrogen and buffered formalin. H&E sections were used to confirm the pathologic diagnosis and the presence of lesional and cancer tissue, verified by a pathologist. Staging of the oral cancer samples was conducted according to American Joint Committee on Cancer (AJCC)/International Union against Cancer (UICC) TNM classification after brief histological studies.

### Immunohistochemistry (IHC)

IHC was performed with various oral tissue samples as described earlier with slight modifications [19]. Briefly, following dewaxing, washing and rehydration of the slides through xylene and graded alcohols, microwave heating in citrate buffer was used for antigen retrieval of GSK3α, GSK3β and pS^9^GSK3β; however for pS^21^GSK3α a high pH flex buffer was used. Endogenous peroxidase was blocked in peroxidase blocking solution (DAKO). Primary antibodies (Santa Cruz Biotechnology) p/GSK3α (dilution 1: 15) and p/GSK3β (dilution 1: 40) were incubated at 4°C overnight. The EnVision FLEX Mini Kit, High pH (Link) (Code: K8023; DAKO) was used for staining. The slides were then washed, and secondary antibody (FLEX-HRP) was applied as dictated by the manufacturer (DAKO Kit). Staining was visualized with diaminobenzidine tetrachloride (DAB). The sections were counterstained with hematoxylin, dehydrated, cleared and mounted. For a negative control, BSA was used in place of the primary antibody. A skin cancer sample known to overexpress GSK3α/β and pGSK3α/β protein was used as a positive control. The normal oral mucosa samples showed moderate immunoreactivity to GSK3α/β and faint immunoreactivity to the pGSK3α/β antibody. Hence, all of the immunostained cancer samples were visualized and scored, such as 0 (no staining), 1 (least intense and staining like normal), 2 (moderately intense staining) and 3 (maximum intense staining) based on the staining intensity and the extent of immunoreactivity. A score of 0 was considered no expression and a score of 1, 2, or 3 was considered for expression of the protein whereas a score of 2 or 3 was considered for the overexpression of the protein.

### Western blot analysis

Western blot analysis was performed as described in detail previously [20]. Cancerous and control tissue lysates were prepared in RIPA buffer (20 mM Tris–HCl pH 7.5; 150 mM NaCl; 1 mM EDTA; 1 mM β-Glycerophosphate; 1% Triton X-100; 2.5 mM Sodium pyrophosphate; 1 mM Sodium orthovanadate; 1 mM PMSF; 0.5% Sodiumdeoxycholate; 10nM Okadoic acid (freshly prepared); 1% SDS; Protease inhibitor (freshly prepared, 1X); Phosphatase inhibitor (freshly prepared, 1X)). Protein samples (60–100 μg) were separated using 10% SDS-PAGE along with a ColourBurst^TM^ Electrophoresis Marker, Sigma (Catalog Number: C1992) and transferred to PVDF membranes using an iBlot^TM^ dry blotting system (BioRad). The blots were cut according to the MW of the protein used for WB analysis. The immunoreactivity of GSK3α was observed much less than GSK3β and therefore a greater amount of tissue extract (TEs) and antibody concentration was used to detect the protein. Immunoblot analysis was performed with the following primary antibodies (Santa Cruz Biotechnology): pGSK3-α (Ser21): sc-101690 (100 μg of resolved TEs, Ab dilution 1: 150); pGSK-3β (Ser9): sc-11757(60 μg of resolved TEs, Ab dilution 1: 300); GSK-3α (H12): sc-5264 (100 μg of resolved TEs, Ab dilution 1: 150); GSK-3β (H-76): sc-9166 (60 μg of resolved TEs, Ab dilution 1: 300); cyclin D1 (DCS-6): sc-20044 (60 μg of resolved TEs, Ab dilution 1: 300), p53(FL-393): sc-6243 (60 μg of resolved TEs, Ab dilution 1: 300) and β-actin (C4): sc-47778 (60 μg of resolved TEs, Ab dilution 1: 1000). The following secondary antibodies (Santa Cruz Biotechnology, dilution 1: 1500), goat anti-rabbit IgG-HRP: sc-2004; rabbit anti-goat IgG-HRP: sc-2768 and donkey anti-mouse IgG-HRP: sc-2314, were used against the respective primary antibody, and the SuperSignal^(R)^ West Pico Chemiluminescent Substrate (Thermo Scientific) was used to detect horseradish peroxidase (HRP) on the immunoblots. A developer, fixer and X-ray film (Kodak) were used to capture the signal.

### Immunoprecipitation and WB

Immunoprecipitation was performed as described previously [21]. Tumor extracts (n = 12) of different stages, T1/T2 (initial stage, n = 6) and T3/T4 (higher stage, n = 6) samples, were used to determine the association of GSK3α/β with cyclin D1. Immunoprecipitation assays were performed using 500 μg of tumor tissue extract (TE), 1 μg GSK3α/β antibody and Protein A/G PLUS-Agarose Immunoprecipitation Reagent (sc-2003, Santa Cruz Biotechnology). The samples were incubated for two hours at RT with shaking and after thorough washing, the immunoprecipitates were run and transferred to PVDF membranes and processed for immunoblotting using a cyclin D1 antibody.

### RT-PCR analysis

Total RNA from 28 samples (6 normal, 6 PMLs and 16 tumor samples) were isolated using the Trizol reagent. The RNA concentration was determined from the OD at a wavelength of 260 nm. The ratio of absorbance at 260 nm and 280 nm was calculated and RNA samples with a ratio of 1.8 to 2.0 were considered pure and included in the study. In total, 5 μg of isolated total RNA was reverse-transcribed to cDNA in a reaction mixture containing 4 μl of 5X reaction buffer, 2 μl of a dNTPs mixture (10 mM), 20 units of an RNase inhibitor, 200 units of an avian-myeloblastosis virus (AMV) reverse transcriptase and 0.5 μg of an oligo(dT) primer (Promega, WI, USA) in a total volume of 20 μl. The reaction mixture was incubated at 42°C for 60 minutes. The reaction was terminated by heating at 70°C for 10 min, and the cDNA was used for RT-PCR. The oligos used for RT-PCR of cyclin D1 were For: 5’ CTC CTG TGC TGC GAA GTG GA 3’; Rev: 5’ AGA CCT CCA GCA TCC AGG TG 3’ and GAPDH were For: 5’ATG GCA AAT TCC ATG GCA CC3’; Rev: 5’ATC CAC AGT CTT CTG GGT GG3’.

### Statistical analysis

The immunostained tissues samples were counted, and a score was given as described and summarized in Tables 1 and 2. Fisher’s exact test and the Chi-square test were used to draw any conclusion. The WB experiments were performed at least in triplicate. The bands were densitometrically analyzed, and the arbitrary units were used for the quantitative expression of various proteins, such as pS^21^GSK3α, pS^9^GSK3β, cyclin D1, and p53. The mean and SD of these arbitrary numbers were used to plot the graphs. Student’s *t*-test was used to compare the differences in various groups. Similarly, the correlation of pS^21^GSK3α and pS^9^GSK3β expression with cyclin D1 and p53 expression of all of the samples were assessed via bivariate analysis using Pearson’s/Spearman’s coefficient. In all of the experiments, a p-value <0.05 was considered statistically significant.

**Table 1:** **Expression of GSK3**α**/GSK3**β **in various OSCC and control samples - their correlation with clinico-pathological parameters**

**Sl. No.**	**Groups**	**Total (N = 80)**	**GSK3α**		**GSK3β**		**p-value (GSK3β** **over GSK3α** **)**	
			**Expression**	**Overexpression**	**Expression**	**Overexpression**	**Expression**	**Overexpression**
**1**	**Age**							
	*≤40*	**15**	**08 (53.3%)**	**00 (00.0%)**	**09 (60.0%)**	**03 (20.0%)**	**NS**	**0.06**
	*>40 ≤ 70*	**53**	**25 (47.1%)**	**05 (09.4%)**	**36 (67.9%)**	**20 (37.7%)**	**0.03**	**0.0006**
	*>70*	**12**	**06 (50.0%)**	**00 (00.0%)**	**09 (75.0%)**	**07 (58.3%)**	**NS**	**0.001**
**2**	**Sex**							
	*Male*	**49**	**26 (53.0%)**	**04 (08.1%)**	**33 (67.3%)**	**19 (38.7%)**	**NS**	**<0.0001**
	*Female*	**31**	**13 (41.9%)**	**01 (03.2%)**	**20 (64.5%)**	**10 (32.2%)**	**NS**	**0.002**
***3***	**Size**							
	*T1-T2*	**40**	**18 (45.0%)**	**03 (07.5%)**	**30 (75.0%)**	**23 (57.5%)**	**0.006**	**<0.0001**
	*T3-T4*	**2**	**02 (100%)**	**00 (00.0%)**	**02 (100%)**	**01 (50.0%)**	**NS**	**NS**
**4**	**Lymph nodes**							
	*N0*	**42**	**19 (45.2%)**	**03 (07.1%)**	**28 (66.6%)**	**20 (47.6%)**	**0.04**	**<0.0001**
	*N1-N3*	**4**	**02 (50.0%)**	**01 (25.0%)**	**03 (75.0%)**	**03 (75.0%)**	**NS**	**NS**
**5**	**Distant Metastasis**							
	*M0*	**38**	**19 (50.0%)**	**03 (07.8%)**	**28 (73.6%)**	**19 (50.0%)**	**0.03**	**<0.0001**
	*M1*	**8**	**03 (37.5%)**	**01 (12.5%)**	**07 (87.5%)**	**07 (87.5%)**	**0.03**	**0.002**
**6**	**Histological grade**							
	*WDSCC*	**24**	**17 (70.8%)**	**02 (08.3%)**	**21 (87.5%)**	**18 (75.0%)**	**NS**	**<0.0001**
	*MDSCC*	**7**	**03 (42.8%)**	**01 (14.2%)**	**07(100%)**	**05 (71.4%)**	**NS**	**NS**
	*PDSCC*	**7**	**02 (28.5%)**	**01 (14.2%)**	**04 (57.1%)**	**03 (42.8%)**	**NS**	**NS**
**7**	**Oral cancer types**							
	*SCC*	**32**	**18 (56.2%)**	**03 (09.3%)**	**27 (84.3%)**	**24 (75.0%)**	**0.02**	**<0.0001**
	*Invasive SCC*	**5**	**02 (40.0%)**	**01 (20.0%)**	**03 (60.0%)**	**03 (60.0%)**	**NS**	**NS**
	*Mucoepidermoid carcinoma*	**8**	**04(50.0%)**	**01 (12.5%)**	**06 (75.0%)**	**03 (37.5%)**	**NS**	**NS**
	*Adamantinoma*	**8**	**03 (37.5%)**	**0 (00.0%)**	**05 (62.5%)**	**01 (12.5%)**	**NS**	**NS**
	*Adenoid cystic carcinoma*	**3**	**00 (00.0%)**	**00 (00.0%)**	**00 (00.0%)**	**00 (00.0%)**	**N/A**	**N/A**
	*Basal cell carcinoma*	**2**	**00 (00.0%)**	**00 (00.0%)**	**01 (50.0%)**	**00 (00.0%)**	**N/A**	**N/A**
	*Acinic cell carcinoma*	**1**	**00 (00.0%)**	**00 (00.0%)**	**01 (50.0%)**	**00 (00.0%)**	**N/A**	**N/A**
**9**	**Hyperplasia of Squamous Epithelium**	**6**	**03 (50.0%)**	**01 (16.6%)**	**04 (66.6%)**	**01 (16.6%)**	**NS**	**NS**
**10**	**Cancer adjacent oral tissue**	**5**	**01 (20.0%)**	**00 (00.0%)**	**03 (60.0%)**	**00 (00.0%)**	**NS**	**NS**
**11**	**Non neoplastic oral cavity glands**	**11**	**07 (63.6%)**	**00 (00.0%)**	**06 (54.5%)**	**00 (00.0%)**	**NS**	**NS**
**12**	**Normal oral squamous epithelium**	**4**	**03 (75.0%)**	**00 (00.0%)**	**01 (25.0%)**	**01 (25.0%)**	**NS**	**NS**
**13**	**Sub-types of OSCC**							
	*Tongue*	**10**	**08 (80.0%)**	**01 (10.0%)**	**08 (80.0%)**	**07 (70.0%)**	**NS**	**0.006**
	*Lip*	**7**	**03 (42.8%)**	**00 (00.0%)**	**07 (100%)**	**07 (100%)**	**0.01**	**0.0002**
	*Cheek*	**6**	**03 (50.0%)**	**00 (00.0%)**	**05 (83.3%)**	**03 (50.0%)**	**NS**	**0.04**
	*Gingiva*	**4**	**02 (50.0%)**	**00 (00.0%)**	**04 (100%)**	**04 (100%)**	**NS**	**0.004**
	*Others*	**5**	**02 (40.0%)**	**03 (60.0%)**	**03 (60.0%)**	**03 (60.0%)**	**NS**	**NS**

**Table 2:** **Expression of pS** ^**21**^ **GSK3**α**/pS** ^**9**^ **GSK3**β **in various OTSCC and control tissue samples and their correlation with clinico-pathological parameters**

**Sl. No.**	**Groups**	**Total (N = 57)**	**pS** ^**21**^ **GSK3α**		**pS** ^**9**^ **GSK3β**		**p-value (pS** ^**9**^ **GSK3β** **over pS** ^**21**^ **GSK3α** **)**	
			**Expression**	**Overexpression**	**Expression**	**Overexpression**	**Expression**	**Overexpression**
**1**	**Age**							
	*≤40*	**8**	**04 (50.0%)**	**03 (37.5%)**	**06 (75.0%)**	**04 (50.0%)**	**NS**	**NS**
	*>40 ≤ 70*	**42**	**29 (69.0%)**	**12 (27.9%)**	**37 (88.0%)**	**30 (71.4%)**	**NS**	**0.0002**
	*>70*	**07**	**05 (71.4%)**	**03 (42.8%)**	**06 (85.7%)**	**05 (71.4%)**	**NS**	**NS**
**2**	**Sex**							
	*Male*	**34**	**22 (64.7%)**	**09 (26.4%)**	**29 (94.5%)**	**22 (64.7%)**	**NS**	**0.0032**
	*Female*	**23**	**16 (69.5%)**	**09 (39.1%)**	**20 (91.3%)**	**17 (73.9%)**	**NS**	**0.036**
**3**	**Histological grade**							
	*WDSCC*	**37**	**29 (78.3%)**	**17 (45.9%)**	**37 (100%)**	**32 (86.4%)**	**0.01**	**0.0004**
	*MDSCC*	**06**	**03 (50.0%)**	**01 (16.6%)**	**06 (100%)**	**02 (33.3%)**	**0.01**	**NS**
	*PDSCC*	**05**	**02 (40.0%)**	**00 (00.0%)**	**03 (60.0%)**	**03 (60.0%)**	**NS**	**NS**
**4**	**Size**							
	T1-T2	**44**	**31 (70.4%)**	**17 (36.1%)**	**41 (93.6%)**	**34 (77.2%)**	**0.01**	**0.0005**
	T3-T4	**06**	**04 (66.6%)**	**01 (16.6%)**	**06 (100%)**	**04 (66.6%)**	**NS**	**NS**
**5**	**Lymph nodes**							
	N0	**47**	**33 (70.2%)**	**18 (38.2%)**	**44 (93.6%)**	**37 (78.7%)**	**0.006**	**0.005**
	N1-N3	**03**	**02 (66.6%)**	**00 (100%)**	**03 (100%)**	**01 (33.3%)**	**NS**	**NS**
**6**	**Distant Metastasis**							
	M0	**50**	**35 (70.0%)**	**18 (36.0%)**	**47 (94.0%**	**38 (76.0%)**	**0.003**	**0.0001**
	M1	**00**	**00 (00.0%)**	**00 (00.0%)**	**00 (00.0%)**	**00 (00.0%)**	**NA**	**NA**
**7**	**Tissue types**							
	*OSCC (Tongue)*	**50**	**35 (70.0%)**	**18 (36.0%)**	**47 (94.0%**	**38 (76.0%)**	**0.003**	**0.0001**
	*Normal Tongue (Cancer adjacent)*	**7**	**03 (42.8%)**	**00 (00.0%)**	**02 (28.5%)**	**01 (14.2%)**	**NS**	**NS**

## Results

### Protein expression of GSK3β is higher than GSK3α in different types of oral tumors

**Figure 1:**
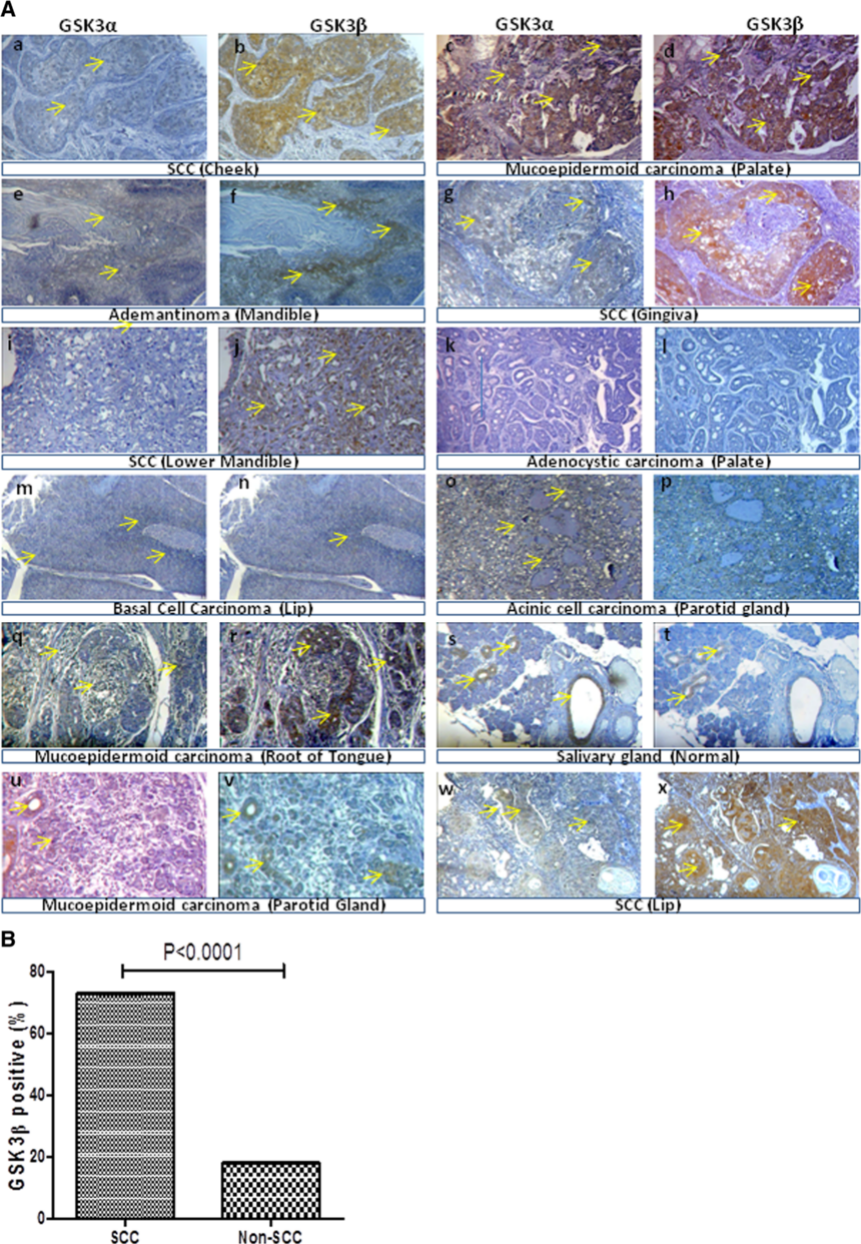
**The expression of GSK3**α **and GSK3β** **proteins in the tumor/ normal tissues of various anatomical sites of the mouth. (A)** Representative immunostaining showing the differential expression of GSK3α and GSK3β from consecutive sections in various types of oral tumor tissue samples as indicated in the figure. (a, b) SCC (cheek); (c, d) Mucoepidermoid carcinoma (palate); (e, f) Adamantinoma (mandible); (g, h) SCC (gingiva); (i, j) SCC (lower mandible); (k, l) Adenoid cystic carcinoma (palate); (m, n) Basal cell carcinoma (lip); (o, p) Acinic cell carcinoma (parotid gland); (q, r) Mucoepidermoid carcinoma (root of the tongue). (s, t) Normal salivary gland, (u, v) Mucoepidermoid carcinoma (parotid gland) (w, x) and SCC (lip) showed differential expression of GSK3α and GSK3β. The maximum intense immunoreactivity of GSK3β was observed in SCC compared to other types of oral tumors. Original magnification 100X. **(B)** Overexpression of GSK3β is significantly higher in SCC than in the other types of (non-SCC) oral cancers (p < 0.0001).

GSK3 immunoreactivity was observed, and different tumors showed the expression of both of the proteins (GSK3α/β) to different extents. GSK3α/β protein expression was observed in the cytoplasmic, nuclear and both the cytoplasmic and nuclear regions of the cancer cells (Figure 1). In most of the samples, intense overexpression of GSK3β compared to GSK3α was observed. The age group >40 ≤ 70 showed expression and overexpression of GSK3β compared to GSK3α, and this observation was statistically significant (p = 0.03 and p = 0.0006, respectively). Similarly, the age group >70 showed more overexpression of GSK3β compared to the GSK3α isoform (p < 0.001). Males and females showed a greater overexpression of GSK3β compared to GSK3α (p < 0.0001 and p = 0.002, respectively). Both expression and overexpression of GSK3β compared to GSK3α was found independently of nodal invasion (p = 0.04 and p < 0.0001). The smaller sized oral tumors showed more expression (p = 0.006) and overexpression (p < 0.0001) of GSK3β compared to GSK3α. Similarly, GSK3β expression and overexpression was significantly higher than GSK3α expression and overexpression in non-metastatic (p = 0.03 & p < 0.0001) and metastatic oral tumors (p = 0.03 & p < 0.002), respectively. All of these independent observations demonstrate a higher expression level of GSK3β than GSK3α in oral tumor tissue samples (Table 1).

### GSK3α/β protein over-expression is significantly associated with OSCC

Various types of oral tumors and control samples (normal squamous epithelium, hyperplasia, cancer adjacent oral tissue, non-neoplastic oral cavity glands, adenoid cystic carcinoma, mucoepidermoid carcinoma, adamantinoma, basal cell carcinoma, OSCC, and acinic cell carcinoma) were analyzed to determine the expression of GSK3α/β by IHC (summarized in Table 1). Normal oral mucosa, hyperplasia and various oral cancer samples showed immunoreactivity to both GSK3α/β antibodies to different extents according to the extent of differentiation but some samples did not show any reactivity. OSCC tissue samples of the cheek (Figure 1. A (a, b)), gingiva (g, h), lower mandible (i, j), and lip (w, x) showed a more intense expression of GSK3β than GSK3α. Similarly, different oral SCC (b, h, j, x) samples showed maximum immunoreactivity to GSK3β. The tissue samples of most of the mucoepidermoid carcinoma showed expression of both GSK3α and GSK3β in ductal cells (c, d and q, r and u, v). The mandibular benign adamantinomas showed immunoreactivity to both GSK3α and GSK3β (e, f). In tissue samples of adenoid cystic carcinoma, staining was not observed, either in ductal or in myoepithelial cells (k, l). The tissue samples of basal cell carcinoma showed very faint expression of both GSK3α and GSK3β (m, n), and acinic cell carcinoma showed no expression (o, p) of either GSK3α or GSK3β. In the normal salivary gland, staining was observed in the ductal cells only and more expression of GSK3α than GSK3β (s, t) was observed. Meanwhile, in total 33.75% (27/80) and 51.25% (41/80) of various oral cancer tissue samples did not show GSK3β and GSK3α protein expression (Table 1), respectively. In addition to OSCC, no significant correlation was observed for various other types of oral cavity neoplasms. The OSCC tumors of various sites, such as tongue, lip, cheek and gingival, showed overexpression of GSK3β compared to GSK3α and was statistically significant (at p = 0.006, p = 0.0002, p = 0.04, p = 0.004, respectively).

The expression of GSK3α was observed more in OSCC tumors than other types of tumors in the oral cavity (such as mucoepidermoid carcinoma, adenoid cystic carcinoma, basal cell carcinoma, adamantinoma, and acinic cell carcinoma considered together) (p = 0.02). Similarly, a significant difference was observed in the overexpression of GSK3β in OSCC tumors compared with other types of tumors as indicated in Table 1 (p < 0.0001; Figure 1B). These results clearly demonstrate a greater role of GSK3β overexpression (which was later found to be mostly inactive) in OSCC.

**Figure 2:**
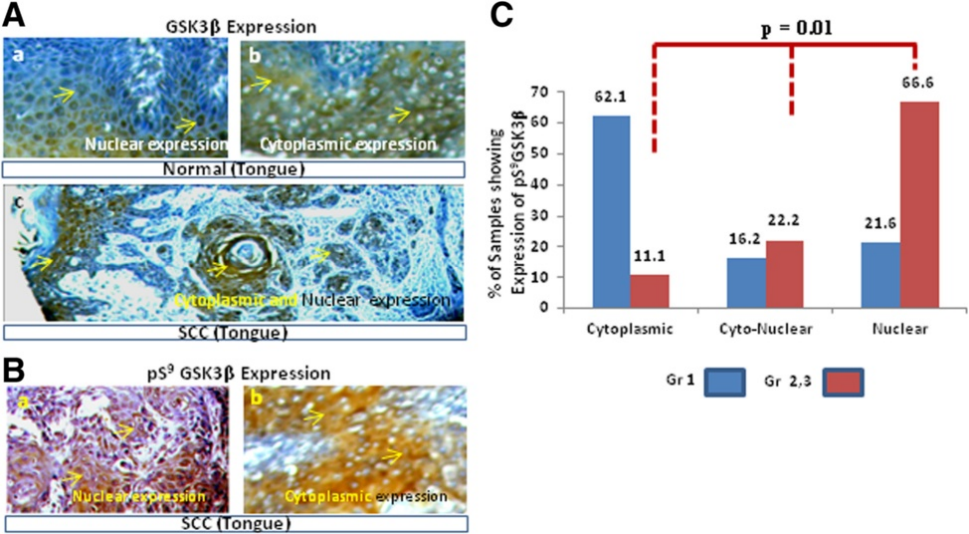
**The expression of active/inactive GSK3**β **proteins in various cellular compartments in normal/OTSCC tissues samples. (A)** The representative photomicrograph shows GSK3β expression in the (a) nuclear compartment (NC) and (b) cytoplasmic compartment (CC) of a normal tongue (200X). (c) GSK3β expression in the nuclear and cytoplasmic compartments (N-CC) in a WDSCC of OTSCC (100X). **(B)** pS^9^GSK3β expression in the (a) cytoplasmic compartment in a MDSCC of an OTSCC sample and (b) the nuclear compartment in a PDSCC of an OTSCC sample. **(C)** A graph showing the percentage of samples of an initial grade (grade-1) or a higher grade (grade 2–3) showing pS^9^GSK3β expression and the shifting of pS^9^GSK3β from the cytoplasm to the nucleus trend in the higher grade samples (p = 0.01).

The cellular expression and distribution of GSK3 was located within different cellular compartments. GSK3β expression was observed in eleven higher grade (MDSCC and PDSCC) tumors. It was expressed in the nuclear compartment (NC), the nuclear-cytoplasmic compartments (N-CC) (Figure 2A (c)) and in only the cytoplasmic compartment (CC) in 5, 4 and 2 cases, respectively. Alternatively, in the lower grade tumors (WDSCC), 4, 5 and 12 samples were found to have positive expression of GSK3β in NC, N-CC and CC, respectively. The trend of GSK3β expression shifting from the cytoplasmic to the nuclear compartment according to disease severity was observed (p = 0.09, though was not significant due to sample size). Alternatively, among the five (5/14) positive tumors of a higher grade (MDSCC and PDSCC), the expression of GSK3α was observed in the NC, N-CC and CC in 2, 2 and 1 of the tumor samples, respectively. Likewise, in the lower grade tumor (WDSCC) samples, GSK3α expression was observed in various cellular compartments, including 5 samples that express it in only the NC, 3 samples in the N-CC and 9 samples in the CC. The normal samples showed expression of GSK3β in the NC and CC in one sample (Figure 2A (a, b)) and the expression of GSK3α in the NC and CC in two and one of the samples, respectively.

### GSK3α and GSK3β protein expression pattern in the progression of OSCC

**Figure 3:**
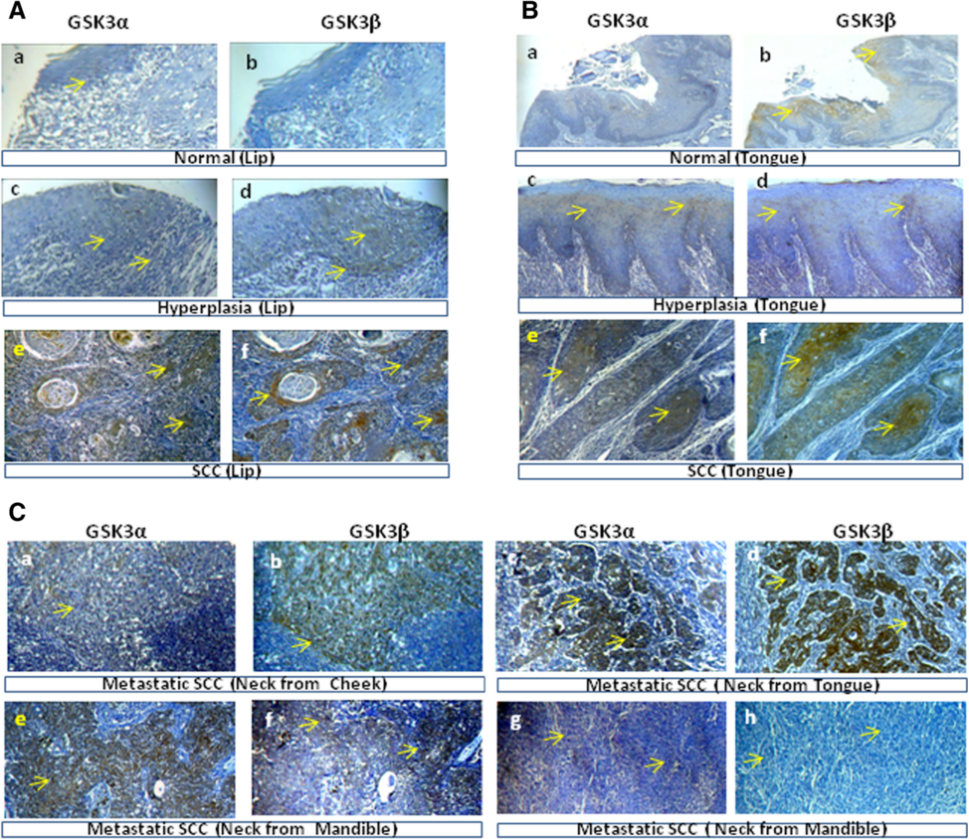
**The expression of GSK3α** **/β** **proteins at various stages of OSCC progression. (A)** Representative immunostaining showing the differential expression of GSK3α and GSK3β from consecutive sections of lip SCC tissue progression, including (a, b) normal lip, (c, d) hyperplasia of the lip and (e, f) SCC of the lip. **(B)** Representative immunostaining showing the differential expression of GSK3α and GSK3β from consecutive sections of OTSCC tissue progression, including (a, b) normal tongue; (c, d) hyperplasia of the tongue and (e, f) SCC of the tongue. **(C)** Photomicrographs showing distant metastasis of SCC cells at the lymph nodes from various OSCC showing immunoreactivity to GSK3α and GSK3β antibodies to different extents in the consecutive sections (a to h). The metastatic OTSCC showed maximum expression of GSK3β (original magnification 100X).

Statistics were gathered to determine the expression of GSK3α and GSK3β in OSCC progression. The results showed that in the normal lip, no immunoreactivity was observed for both the GSK3α and the GSK3β antibody (Figure 3A (a, b)). However, in the normal tongue tissue, mild expression of the GSK3α/β protein was observed in the peripheral epithelial layer in most of the cases (Figure 3B (a, b)), except one sample that showed strong immunoreactivity to the GSK3β antibody. In the tissue samples with mild hyperplasia, the expression of GSK3α and GSK3β was observed, and it was limited to the deeper epithelial zone of both the lip and tongue tissues (Figure 3A (c, d) & B (c, d)). Alternatively, the expression of the GSK3α/β protein was observed in the tumors of the lip and tongue (Figure 3A (e, f) & B (e, f)). In the OTSCC progression model, a total of twenty-two samples were analyzed, and GSK3β overexpression was observed in 14.2% (1/7) of normal samples, in 20.0% (1/5) of hyperplasia and 70.0% (7/10) of cancer samples (p = 0.0396). In the lip cancer progression model, a total of fifteen samples were analyzed and GSK3β overexpression was not observed in normal (n = 2) or hyperplasia (n = 3) samples but was observed in 70.0% (7/10) of cancer samples (p = 0.0376). In the distant metastatic SCC samples, the invasive cancer cells at the new location demonstrated an overexpression of GSK3β in 87.5% (7/8) whereas only 12.5% (1/8) of samples showed an overexpression of GSK3α (p = 0.002) (Table 1 and Figure 3C (a to h)). Similarly, nearby lymph node positive cases were found in 60% (3/5) of the GSK3β overexpressing tumors and in only 20% (1/5) of the GSK3α overexpressing tumors.

### Progressive inactivation of the GSK3α/β protein expression in the OTSCC

**Figure 4:**
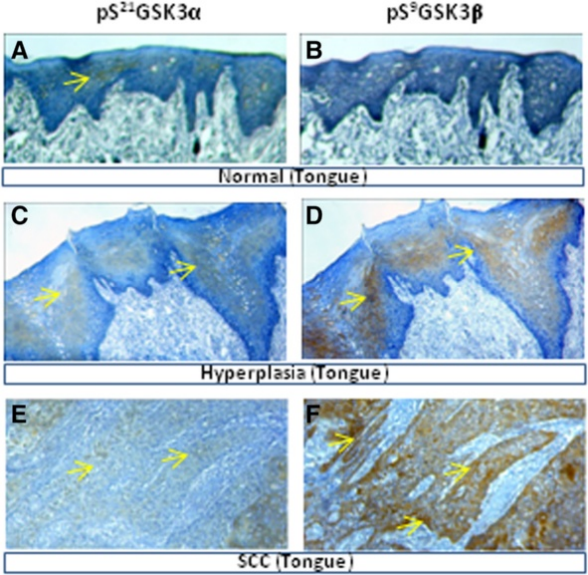
**The inactivation of GSK3 proteins at various stages of OTSCC progression.** Representative immunostaining showing the differential expression of pS^21^GSK3α and pS^9^GSK3β in the consecutive sections of **(a, b)** normal tongue tissue, **(c, d)** mild hyperplasia of tumor adjacent tongue, and **(e, f)** OTSCC tissue samples (original magnification 100X).

Inactivation of the GSK3 proteins was detected by determining the expression of pS^21^GSK3α and pS^9^GSK3β at various stages in OTSCC progression. Cancer adjacent apparently normal tongue samples showed faint immunoreactivity of pS^21^GSK3α and pS^9^GSK3β in 42.8% (3/7) and 28.5% (2/7) of the samples (Figure 4. a, b), respectively. However, the overexpression of pS^9^GSK3β was not observed in 85.7% (6/7) of cancer adjacent normal looking tongue samples. One tumor adjacent tongue sample with mild hyperplasia showed moderate expression of both pS^21^GSK3α and pS^9^GSK3β (Figure 4. c, d). Alternatively, in total 85.96% (49/57) and 66.6% (38/57) of OTSCC tissue samples showed pS^9^GSK3β and pS^21^GSK3α protein expression, respectively (Figure 4 e, f) (Table 2). Moreover, 80% (8/10) of the OTSCC samples showed immunoreactivity to GSK3α and GSK3β antibodies (Table 1). If this statistic remains consistent, then all of the OTSCC samples that express GSK3β may be inactivated and nearly 15.0% of all of the OTSCC samples that express GSK3α may still remain active.

Both male and female patient tissue samples showed overexpression of pS^21^GSK3α (p = 0.0032) and pS^9^GSK3β (p = 0.036). Meanwhile, in the age group >40 ≤ 70, the over-expression of inactive GSK3β compared to GSK3α was observed (p = 0.0002). Similarly, the extent of inactive GSK3β expression was observed more than GSK3α expression in small sized (T1-T2 group) tumors (p = 0.0005). The over-expression of the pS^9^GSK3β protein was observed in 86.4% of WDSCC (32/37), 33.3% of MDSCC (2/6) and 60.0% of PDSCC (3/5) samples (p = 0.01). Alternatively, the expression of the pS^9^GSK3β protein was observed in all of the WDSCC and MDSCC (43/43) and observed in at least 60.0% of the PDSCC (3/5) samples (p = 0.0001).

### Nuclear accumulation of pGSK3α/β protein expression in the OTSCC

The distribution of pS^9^GSK3β was observed in the nuclear, cytoplasmic or both cellular compartments in human OTSCC samples (Figure 2B (a, b)). Among the nine pS^9^GSK3β-expressing tumors of a higher grade (MDSCC and PDSCC), pS^9^GSK3β expression was observed in the NC, N-CC and in only CC in 6, 2 and 1 samples, respectively. Alternatively, in the lower grade tumors (WDSCC), 8, 6 and 23 samples showed pS^9^GSK3β expression in the NC, N-CC and in only CC, respectively (Figure 2B (a, b)). This trend of pS^9^GSK3β expression shifting from the cytoplasmic to the nuclear compartment with tumor progression was significant (p = 0.01; Figure 2C). Alternatively, a faint reactivity of pS^21^GSK3α was observed in the OTSCC samples. Among the pS^21^GSK3α-stained five positive tumors of a higher grade (MDSCC and PDSCC), the expression in the NC, N-CC and only CC was observed in 3, 1 and 1 tumor samples, respectively. Similarly, in the lower grade tumors (i.e., WDSCC), 4, 7 and 18 tumor samples were positive for pS^21^GSK3α in the NC, N-CC and in only CC, respectively (p = 0.054).

### Inactivation of GSK3α/β and their correlation with cyclin D1 and p53 in human OSCC

**Figure 5:**
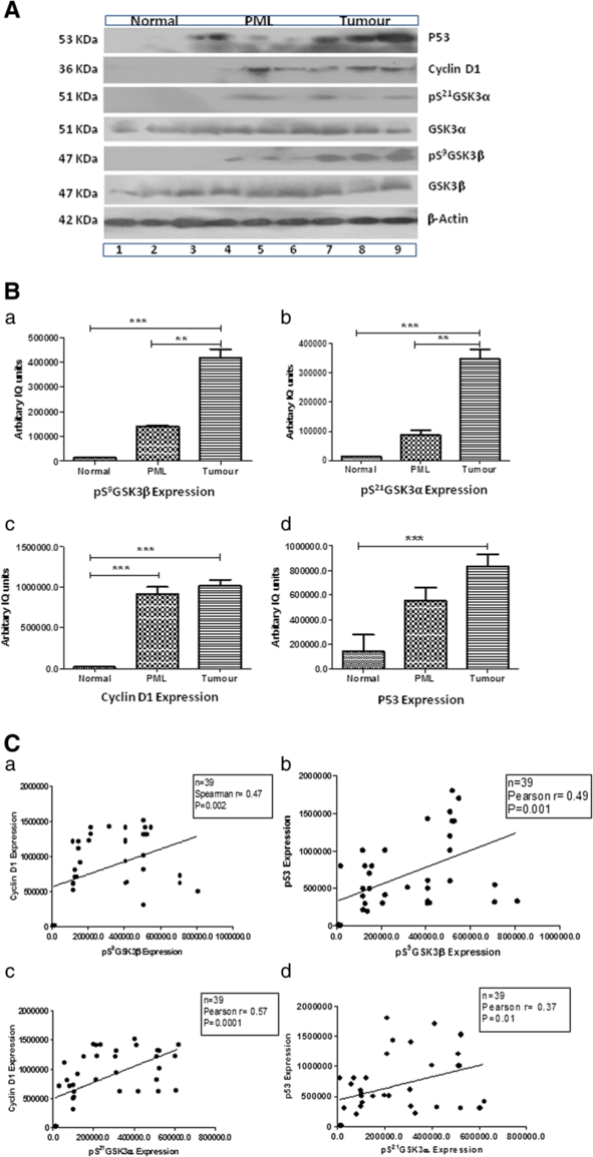
**WB analysis to show the expression of in/active GSK3, cyclin D1 and p53 proteins at various stages of OSCC. (A)** Representative blot showing the expression of GSK3α, GSK3β, pSer^21^GSK3α, pSer^9^GSK3β, cyclin D1 and p53 in various normal, PMLs and OSCC samples. β-Actin was used as a loading control in this experiment. **(B)** The mean and SD of each protein band has been plotted for the normal, PML and OSCC samples. Statistical analysis was performed using Student’s *t*-test. Statistical significance was observed among various groups: **p = 0.001, ***p < 0.0001 as indicated in the figure. The comparison of (a) pS^9^GSK3β, (b) pSer^21^GSK3α, (c) cyclin D1, and (d) p53 protein expression among the groups of samples. **(C)** A positive correlation was obtained in different pairs: (a) pSer^9^GSK3β and cyclin D1 (p = 0.002), (b) pSer^21^GSK3α and cyclin D1 (p = 0.001), (c) pSer^21^GSK3α and p53 (p = 0.0001), and (d) pSer^9^GSK3β and p53 (p = 0.01).

The expression of GSK3α/β, cyclin D1 and p53 (n = 39; Table 3) was detected using WB analysis. Detectable expression of GSK3α was observed in 83.3% normal (5/6), 66.6% PMLs (4/6), and 51.8% tumor samples (14/27). Similarly, the expression of GSK3β was observed in 83.3% normal (5/6), 83.3% PMLs (5/6) and 59.25% oral tumor (16/27) samples. Moreover, 25.9% (7/27) and 40.7% (11/27) of oral tumor samples showed decreased expression of GSK3α and GSK3β expression compared to normal samples. The expression of pSer^21^GSK3α and pSer^9^GSK3β was not observed in normal samples and less expression was observed in the PMLs samples. Alternatively, 81.4% (22/27) and 85.1% (23/27) of the OSCC samples showed immunoreactivity for pSer^21^GSK3α and pSer^9^GSK3β, respectively. Overexpression of the cyclin D1 protein was observed in 70.3% (19/ 27) of OSCC samples and in 100% (6/6) of PMLs compared to the normal oral mucosa tissue samples. Expression of the p53 protein was observed in 48.1% (13/ 27) of the OSCC samples, 50.0% (3/6) of the PMLs samples and 16.6% (1/6) of the normal oral mucosa samples. β-Actin was used as a loading control in these experiments (Figure 5A).

**Table 3:** **Patient characteristics and expression of pS** ^**21**^ **GSK3**α**, pS** ^**9**^ **GSK3**β**, cyclin D1 and P53 in oral cancer and control tissue samples**

**Sl. No.**	**Groups**	**Total (n = 39)**	**Samples showing the positive expression of proteins (n)**			
			**Cyclin D1**	**P53**	**pS** ^**21**^ **GSK3α**	**pS** ^**9**^ **GSK3β**
**1**	**Age**					
	*≤40*	**23**	**17 (73.9%)**	**08 (34.7%)**	**12 (52.1%)**	**14 (60.8%)**
	*>40 ≤ 70*	**16**	**08 (50.0%)**	**08 (50.0%)**	**10 (62.5%)**	**09 (56.2%)**
**2**	**Sex**					
	Male	**23**	**14 (60.8%)**	**10 (43.4%)**	**13 (56.5%)**	**14 (60.8%)**
	Female	**16**	**11 (68.7%)**	**06 (37.5%)**	**09 (56.2%)**	**09 (56.2%)**
**3**	**Histological grade/Tumour Progression**					
	Normal	**6**	**00 (00.0%)**	**01 (16.6%)**	**00 (00.0%)**	**00 (00.0%)**
	PML	**6**	**06 (100%)**	**03 (50.0%)**	**01 (16.6%)**	**02 (33.3%)**
	WDSCC	**15**	**10 (66.6%)**	**07 (46.6%)**	**10 (66.6%)**	**11 (73.3%)**
	MDSCC	**6**	**06 (100%)**	**05 (83.3%)**	**06 (100%)**	**05 (83.3%)**
	PDSCC	**6**	**03 (50.0%)**	**01 (16.6%)**	**05 (83.3%)**	**05 (83.3%)**
**4**	**Tobacco History**					
	Yes	**23s**	**14 (60.8%)**	**11 (47.8%)**	**12 (52.1%)**	**13 (56.5%)**
	No	**16**	**11 (68.7%)**	**05 (31.2%)**	**10 (62.5%)**	**10 (62.5%)**
**5**	**pS** ^**21**^ **GSK3α** **Expression**					
	Positive	**22**	**18 (81.8%)**	**12 (54.5%)**	**22 (100%)**	**20 (90.9%)**
	Not-Positive	**17**	**07 (41.1%)**	**04 (23.5%)**	**00 (00.0%)**	**03 (17.6%)**
**6**	**pS** ^**9**^ **GSK3β** **Expression**					
	Positive	**23**	**18 (78.2%)**	**11 (47.8%)**	**20 (86.9%)**	**23 (100%)**
	Not Positive	**16**	**07 (43.7%)**	**05 (31.2%)**	**02 (12.5%)**	**00 (00.0%)**

The expression of pS^9^GSK3β (p = 0.001) and pS^21^GSK3α (p = 0.0001) was significantly different in tumors compared to normal and PMLs (Figure 5B (a and b)). Similarly, cyclin D1 protein expression was observed more in tumor samples and PML samples than in normal samples (p = 0.0001; Figure 5B (c)). The overexpression of the p53 protein was greater in oral tumor samples than in the normal counterpart (p = 0.0001; Figure 5B (d)). Further, the expression of pS^21^GSK3α and pS^9^GSK3β was positively correlated with cyclin D1 expression (p = 0.0001 and p = 0.002, respectively; Figure 5C (a, b)). Similarly, p53 expression was positively correlated with the expression of pS^21^GSK3α and pS^9^GSK3β (p = 0.01 and p = 0.001, respectively; Figure 5C (c, d)).

### The interaction of GSK3α/β with cyclin D1 in human OSCC

**Figure 6:**
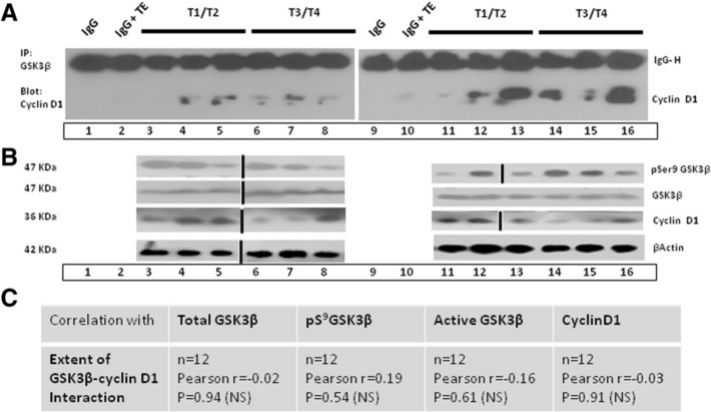
**The interaction of GSK3β** **with cyclin D1 in different stages oral cancer progression. (A)** The interaction of GSK3β with cyclin D1 in various oral tumor extracts (T1-T2 TE lane 3–5 & 11–13 and T3-T4 TE in lane 6–8 and 14–16). The input IgG and IgG adsorbed in TEs served as controls. **(B)** The expression levels of GSK3β, pSer^9^GSK3β, cyclin D1 and β-actin in the TEs were checked in the TEs used for interaction studies. **(C)** No significant correlation was observed in the interaction of GSK3β-cyclin D1 with the expression of various proteins as indicated.

The interaction of GSK3β and cyclin D1 was observed in various oral tumor samples (Figure 6A). Alternatively, a GSK3α-cyclin D1 interaction was not observed. A GSK3β-cyclin D1 association was observed in 100% (6/6) of the higher stage (T3/T4) tumors compared to 66.6% (4/6) of the initial stage (T1/T2) oral tumors samples. The expression of pS^9^GSK3β, total GSK3β and cyclin D1 was detected in the corresponding WCE (Figure 6B) and was correlated with the extent of the GSK3β-cyclin D1 interaction. The results show no statistically significant correlation between the extent of the GSK3β-cyclin D1 interaction and the level of expression of total GSK3β, pS^9^GSK3β, and cyclin D1. Moreover, no correlation was observed between the extent of the interaction and the active fraction of GSK3β (arbitrary units of the GSK3β reading minus the pS^9^GSK3β reading) as shown in Figure 6C.

### Correlation of GSK3α/β inactivation status with cyclin D1 transcription in human OSCC

**Figure 7:**
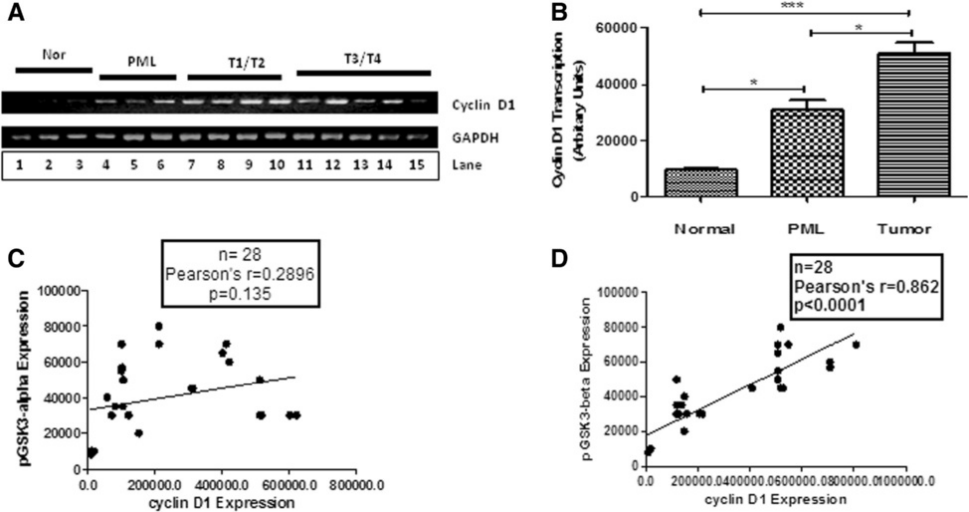
**The correlation of pSer** ^**21**^ **GSK3α** **/pSer** ^**9**^ **GSK3β** **expression with cyclin D1 transcription in various OSCC samples. (A)** RT-PCR showing cyclin D1 mRNA expression (201 bp PCR product) in different normals (lane 1–3), PMLs (lane 4–6) and oral cancer samples of various stages (T1-T2 samples lane 7–10; T3-T4 samples lane 11–15). GAPDH expression (410 bp PCR product) was used as a control in this experiment. **(B)** A histogram showing the level of expression of cyclin D1 mRNA in various groups (N-Normal, PML, T-Tumor) of samples as indicated. No significant correlation **(C)** of cyclin D1 mRNA expression and pSer^21^GSK3α protein expression was observed, and a positive correlation **(D)** between cyclin D1 mRNA expression and the expression of pSer^9^GSK3β was observed.

RT-PCR analysis was performed to determine the expression of cyclin D1 mRNA in various tumor, PML and normal samples (Figure 7A). PMLs and tumor samples showed increased cyclin D1 mRNA levels compared to normal oral mucosa (Figure 7B). The mRNA expression was correlated with the protein expression in the same tissue samples. The correlation of pS^21^GSK3α expression with cyclin D1 mRNA expression was not significant (n = 28, Pearson’s r = 0.2896, p = 0.135) whereas the correlation of pS^9^GSK3β expression with cyclin D1 mRNA expression was significant (n = 28, Pearson’s r = 0.8624, p < 0.0001; Figure 7C and D).

## Discussion

The deregulation of GSK3 is involved in several types of human cancer and neurodegenerative diseases [7,22]. It has two isoforms, GSK3α and GSK3β, and their expression varies in different tissue types [23]. To the best of our knowledge, no reports are available regarding the variation of both isoforms (GSK3α and GSK3β) in human mouth cancer. The present study revealed an increased protein expression of GSK3β compared to GSK3α in various types of mouth neoplasms. Although the overexpression of GSK3β and the mild expression of GSK3α were found in various types of cancer and benign tumors of the mouth, the expression was mainly detected in OSCC. Mucoepidermoid carcinoma and normal salivary glands exhibited expression of GSK3α and GSK3β, mainly in the ductal cells. The expression of total GSK3α/β in the tumor samples generally increased from the normal expression level but a small fraction of the tumor tissue also showed the opposite trend. Previously, GSK3β expression has been correlated with a favorable outcome in OTSCC [24]. We also observed very high overexpression of GSK3β in the tongue tissue samples of normal, benign, malignant and even metastatic cancers. There have been a number of conflicting reports concerning the extent of tumor progression and the expression of total GSK3β in human cancers [25,26]. In the present study, we found that GSK3β expression plays a key role in oral cancer. The cause may be that the major pool of total GSK3 is inactivated, which was consistent with our earlier report on DMBA-induced hamster cheek pouch carcinomas [19].

The site-specific phosphorylation of pS^21^GSK3α and pS^9^GSK3β residues changes their activity and makes them catalytically inactive [10]. Because we have observed more GSK3s in the tongue samples, its inactivation status was also detected. The expression level of pS^21^GSK3α and pS^9^GSK3β steadily increased from normal to hyperplasia to benign tumors to carcinomas, indicating that there is an active role of inactive GSK3α/β in OTSCC progression. The inactivation of GSK3β was reported in tongue cancer [27,28]. To the best of our knowledge, this is the first report showing the inactivation of GSK3α in OTSCC. Our present study provides evidence that the progressive inactivation of GSK3α/β is a common event in human OTSCC.

The experimental results provide evidence of a decrease in GSK3α/β levels in some OSCC tumors compared to normal. This observation may be due to IHC staining, either by over fixation of the tissue samples or silencing of GSK3α/GSK3β. The latter seems to be true because some of the fresh tissue samples showed decreased reactivity to the GSK3α/β antibody. This result may be due to the deregulation of transcription, reduced mRNA stability or rapid protein turnover. Further investigation is warranted to investigate the mechanism of down regulation of GSK3α/β in certain fractions of oral cancer. There seems to be alternative mechanisms that inactivate GSK3α/β, leading to silencing, to promote OSCC.

The protein expression of GSK3α/β and phospho-GSK3α/β was detected in different cellular compartments (such as NC, N-CC and CC). A higher expression of pS^9^GSK3β was observed in the nuclear compartments in the higher grade OTSCC (p = 0.01). Though the correlation of the expression of p^21^GSK3α with higher grade samples was not statistically significant, a similar trend was observed. Alternatively, in OTSCC, cytoplasmic expression of pS^9^GSK3β and p^21^GSK3α was associated with low grade histology. Hence, the pGSK3α/β protein accumulates and/or translocates from the cytoplasm to the nucleus to a greater extent as OTSCC progresses. With regard to the tumor adjacent (apparently normal) tissues and hyperplasia that displayed no staining or faint staining of pS^9^GSK3β and pS^21^GSK3α in the cytoplasmic regions, it seems that inactive GSK3α/β expression in the cytoplasm contributes to tumor progression whereas nuclear expression of inactive GSK3α/β leads to a more severe disease. This finding may have predictive value for OTSCC and its outcome.

Cyclin D1 has been established as a potent proto oncogene, and the overexpression of cyclin D1 has been observed frequently in human cancer, including OSCC [29]. Cyclin D1 turnover is dependent on threonine^286^ phosphorylation, and active GSK3β was also shown to promote this event [14]. In this context, the positive correlation of inactive GSK3α/β with cyclin D1 is encouraging. The robust interaction of GSK3β with cyclin D1 was observed in oral tumor samples. We were unable to detect whether the active or inactive fraction of GSK3β interacted with cyclin D1 due to technical difficulties (antibody heavy chain interferences). Surprisingly, the advanced stage tumors showed a greater interaction of GSK3β with cyclin D1, which was counterintuitive to our finding of progressive inactivation of GSK3β. Moreover, a significant correlation was observed when inactive GSK3β expression and cyclin D1 mRNA expression was compared. This observation may be due to the activation of some downstream TFs of GSK3β to fuel cyclin D1 expression and boost the uncontrolled cell division program in OSCC.

p53 is the guardian of the genome and a well-known tumor suppressor, and its loss of function is the most frequent genetic event in human cancer [30,31]. Although it is inactivated by a number of pathways, GSK3β is a key regulatory molecule of p53 [32]. GSK3 has been reported to phosphorylate p53 on Ser^33^-p53 or Ser^315^-p53 and Ser^376^-p53 and promote the acetylation of p53, thus controlling the function of p53 [18]. There are numerous studies on p53 expression in OSCC that are inconclusive [33]. However, the results of the present study indicate that p53 expression is higher in the subset of oral tumors with inactive GSK3. A positive correlation was observed between inactive GSK3 and p53 expression. Inactive GSK3 as a result of number of major signaling pathways including the phosphatidyl-inositol-3-kinase (PI3K) pathway, the Wnt pathway, Hedgehog signaling and Notch signaling, may modulate the status of many oncogenic TFs, RNA-binding proteins, miRNAs, and might be the cause of increased p53 expression in OSCC [34-36]. The exact molecular mechanism of GSK3β mediated p53 expression remains unexplored. Moreover, in parallel with the oral tumor progression, the nuclear accumulation of pS^9^GSK3β was observed, and this observation may be an impediment to activate p53 and may restrict uncontrolled cell division.

**Figure 8:**
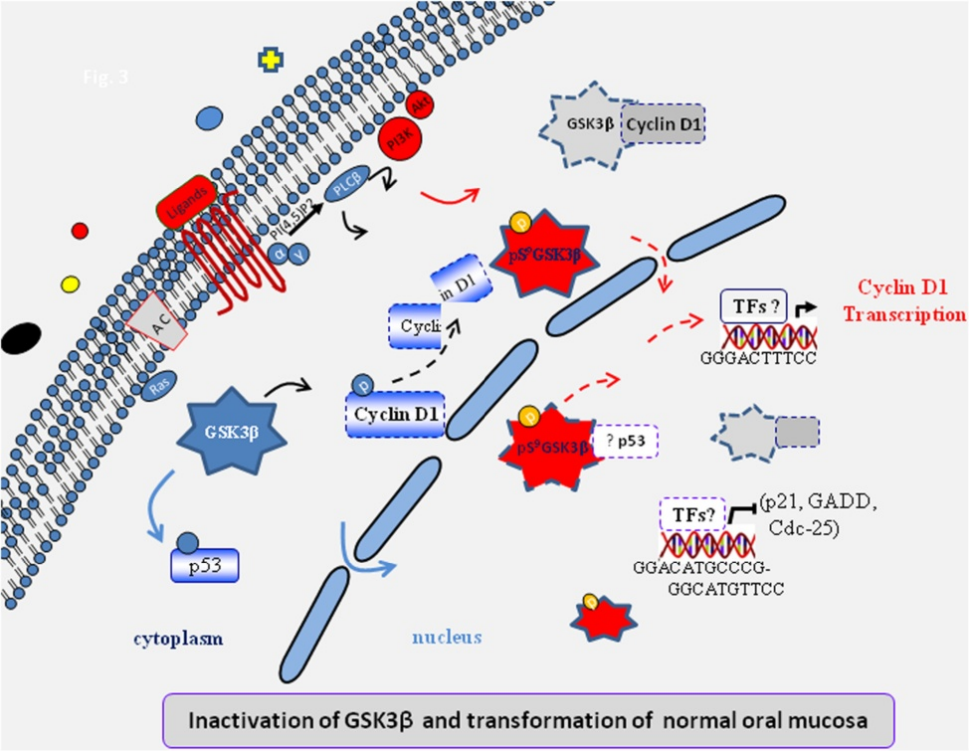
**A proposed model for defining GSK3-mediated deregulation of cell division in oral cancer.** Diverse upstream signaling pathways and regulatory molecules contribute to the loss of function of GSK3 by reversible phosphorylation, inactivating GSK3. These functionally altered GSK3 may shift to various cellular compartments, fuel cyclin D1 transcription possibly by increasing the activity of various TFs or may remain silent, due to degrading cyclin D1 or activating p53, to transform the oral mucosa leading to OSCC.

Finally, growth factor stimulation and oncogenic transformation may lead to increased glucose metabolism in the transformed oral mucosa that may increase the pool of inactive GSK3. This inactive GSK3 (mainly GSK3β) may initiate a signaling mechanism that promotes transcriptional activation of cyclin D1 by targeting some yet unknown TFs to fuel OSCC. Moreover, the inhibition of kinase activity and the shift in cellular compartments may affect the subpopulation of p53 that is not inactivated by a mutation (Figure 8). Our study strongly suggests that GSK3 expression may be used as a molecular marker for the diagnosis and therapeutic intervention of OSCC.

## Conclusion

In summary, although increased expression of both GSK3α and GSK3β was associated with human oral tumor pathogenesis, progressive inactivation of GSK3β was observed in OSCC particularly in OTSCC. A positive correlation was observed between expression of pS^9^GSK3β and cyclin D1 protein expression, p53 protein expression and cyclin D1 mRNA expression in human oral cancer/control tissue samples. Hence, the expression of pS^9^GSK3β can be used as a marker for assessing disease severity. Further research is warranted to elucidate the additional mechanisms involved so as to develop appropriate therapeutic interventions.
