# Correction: Expression and inactivation of glycogen synthase kinase 3 alpha/ beta and their association with the expression of cyclin D1 and p53 in oral squamous cell carcinoma progression

**DOI:** 10.1186/s12943-024-02058-z

**Published:** 2024-07-25

**Authors:** Rajakishore Mishra, Siddavaram Nagini, Ajay Rana

**Affiliations:** 1 Centre for Life Sciences, School of Natural Sciences, Central University of Jharkhand, Ratu-Lohardaga Road, Brambe, Ranchi, Jharkhand 835205 India; 2 Department of Biochemistry and Biotechnology, Faculty of Science, Annamalai University, Annamalainagar, Tamil Nadu 608 002 India; 3 Department of Molecular Pharmacology & Therapeutics, Loyola University Chicago, 2160 South First Ave., Maywood, 60153IL USA

**Correction: Mol Cancer 14, 20 (2015)**

**https://doi.org/10.1186/s12943-015-0300-x**

Following publication of the original article [1], the authors learned that an incorrect representative IHC image (Fig. 1n) for GSK3beta was used. We have identified the error and would like to replace it with the corrected Fig. 1n. The image replacement does not alter the paper's conclusions, and our proposed hypothesis remains the same. They apologize for this oversight.

The incorrect Fig. 1:

**Fig. 1:**
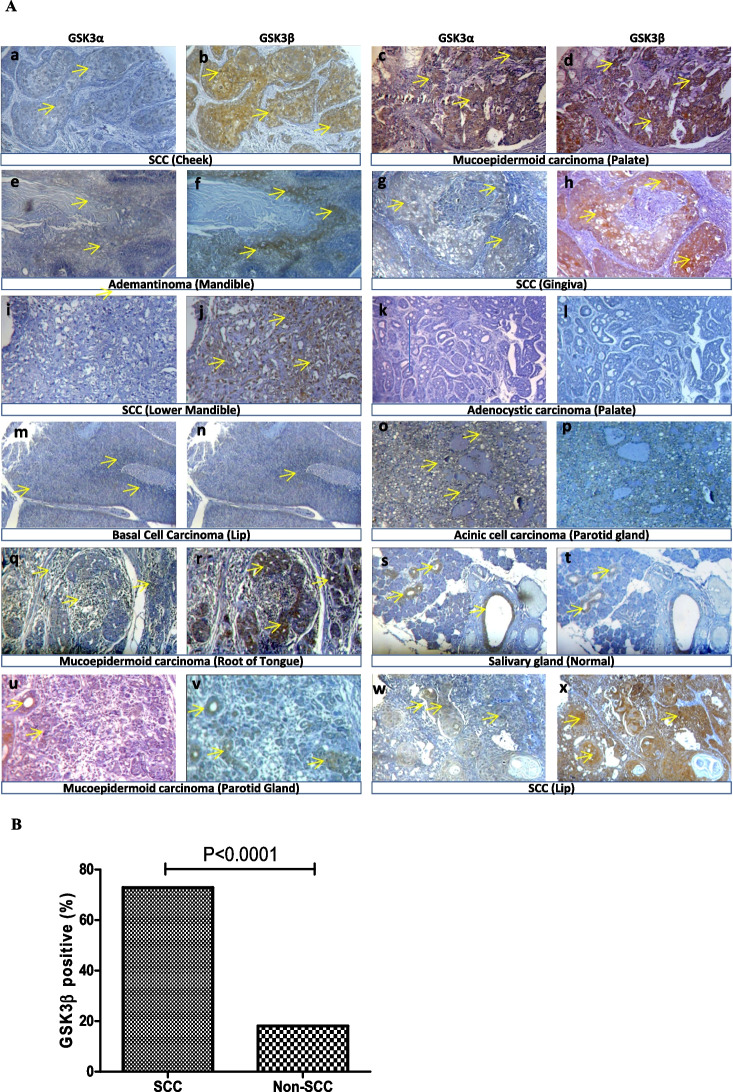
**The expression of GSK3**α **and GSK3β** **proteins in the tumor/ normal tissues of various anatomical sites of the mouth. (A)** Representative immunostaining showing the differential expression of GSK3α and GSK3β from consecutive sections in various types of oral tumor tissue samples as indicated in the figure. (a, b) SCC (cheek); (c, d) Mucoepidermoid carcinoma (palate); (e, f) Adamantinoma (mandible); (g, h) SCC (gingiva); (i, j) SCC (lower mandible); (k, l) Adenoid cystic carcinoma (palate); (m, n) Basal cell carcinoma (lip); (o, p) Acinic cell carcinoma (parotid gland); (q, r) Mucoepidermoid carcinoma (root of the tongue). (s, t) Normal salivary gland, (u, v) Mucoepidermoid carcinoma (parotid gland) (w, x) and SCC (lip) showed differential expression of GSK3α and GSK3β. The maximum intense immunoreactivity of GSK3β was observed in SCC compared to other types of oral tumors. Original magnification 100X. **(B)** Overexpression of GSK3β is significantly higher in SCC than in the other types of (non-SCC) oral cancers (p < 0.0001)

The correct Fig. 1:

**Fig. 1:**
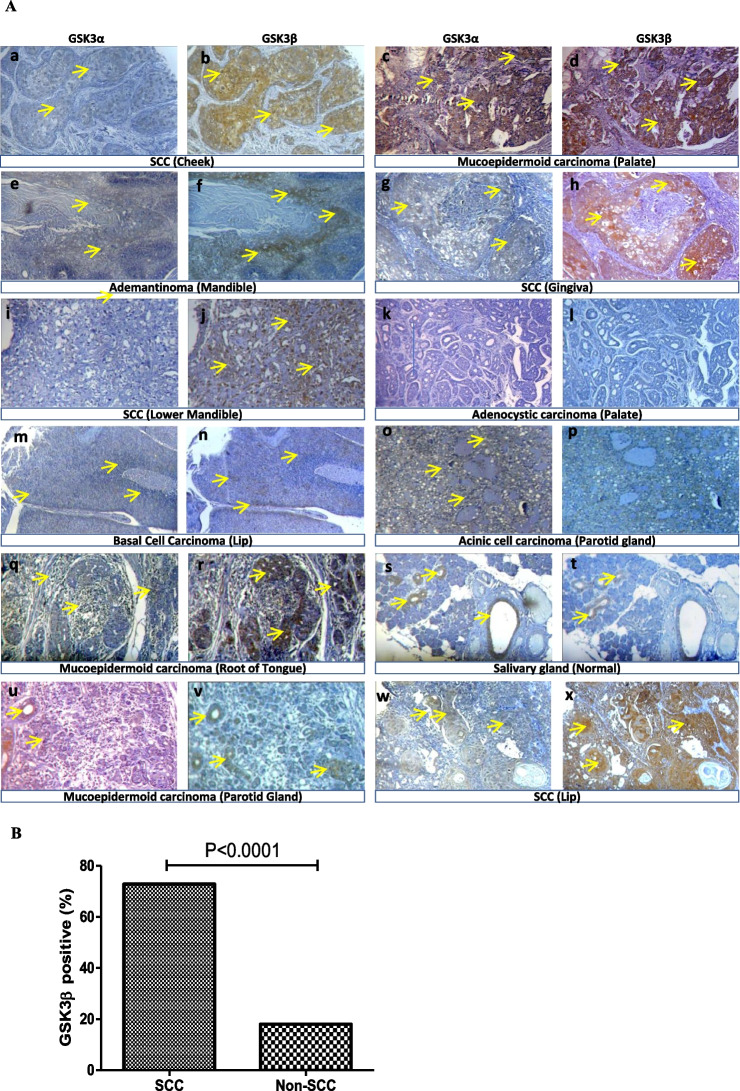
**The expression of GSK3**α **and GSK3β** **proteins in the tumor/ normal tissues of various anatomical sites of the mouth. (A)** Representative immunostaining showing the differential expression of GSK3α and GSK3β from consecutive sections in various types of oral tumor tissue samples as indicated in the figure. (a, b) SCC (cheek); (c, d) Mucoepidermoid carcinoma (palate); (e, f) Adamantinoma (mandible); (g, h) SCC (gingiva); (i, j) SCC (lower mandible); (k, l) Adenoid cystic carcinoma (palate); (m, n) Basal cell carcinoma (lip); (o, p) Acinic cell carcinoma (parotid gland); (q, r) Mucoepidermoid carcinoma (root of the tongue). (s, t) Normal salivary gland, (u, v) Mucoepidermoid carcinoma (parotid gland) (w, x) and SCC (lip) showed differential expression of GSK3α and GSK3β. The maximum intense immunoreactivity of GSK3β was observed in SCC compared to other types of oral tumors. Original magnification 100X. **(B)** Overexpression of GSK3β is significantly higher in SCC than in the other types of (non-SCC) oral cancers (p < 0.0001)
